# Health disparities of critically ill children according to poverty: the Korean population-based retrospective cohort study

**DOI:** 10.1186/s12889-021-11324-4

**Published:** 2021-06-30

**Authors:** Esther Park, Hyejeong Park, Danbee Kang, Chi Ryang Chung, Jeong Hoon Yang, Kyeongman Jeon, Eliseo Guallar, Juhee Cho, Gee Young Suh, Joongbum Cho

**Affiliations:** 1 Department of Critical Care Medicine, Samsung Medical Center, Sungkyunkwan University School of Medicine, 81 Irwon-ro, Gangnam-gu, Seoul, 06351 Republic of Korea; 2 Center for Clinical Epidemiology, Samsung Medical Center, Seoul, Republic of Korea; 3 Department of Clinical Research Design & Evaluation, SAIHST, Sungkyunkwan University, Seoul, Republic of Korea; 4 Department of Epidemiology, Welch Center for Prevention, Epidemiology, and Clinical Research, Johns Hopkins Bloomberg School of Public Health, Baltimore, MD USA; 5 Department of Medicine, Welch Center for Prevention, Epidemiology, and Clinical Research, Johns Hopkins Bloomberg School of Public Health, Baltimore, MD USA

**Keywords:** Health disparity, Critical care, Poverty, Intensive care units, Mortality, Child, National health insurance, Patient admission, Patient readmission, Health status

## Abstract

**Background:**

There is a lack of nationwide studies on critically ill patients’ health disparity under the National Health Insurance (NHI) system. We evaluated health disparities in intensive care unit (ICU) admission, outcomes, and readmission in impoverished children.

**Methods:**

We conducted a retrospective cohort study using a national database from the Korean NHI and Medical Aid Program (MAP). MAP supports the population whose household income is lower than 40% of the median Korean household income. We defined poverty as being a MAP beneficiary and compared the poverty and non-poverty groups. Patients between 28 days and 18 years old who were admitted to the ICU were included. Hospital mortality and readmission were analyzed with adjustment for patient characteristics, hospital type, and management procedures.

**Results:**

Out of 17,893 patients, 1153 (6.4%) patients were in poverty. The age-standardized ICU admission rate was higher in the poverty group (126.9 vs. 80.2 per 100,000 person-years). There was more age-standardized mortality in the poverty group (11.8 vs. 4.3 per 100,000 person-years). Patients in the poverty group did not have a statistically different risk of adjusted in-hospital mortality to those in the non-poverty group (odds ratio: 1.15, confidence interval [CI]: 0.84–1.55) but had a higher readmission rate (hazard ratio 1.25, CI 1.09–1.42).

**Conclusion:**

Under the NHI system, the disparity in pediatric critical care outcomes according to poverty is not definite, but the healthcare disparity in pre- and post-hospital care is a concern. Further studies are required to improve pre- and post-hospital healthcare quality of impoverished children.

**Supplementary Information:**

The online version contains supplementary material available at 10.1186/s12889-021-11324-4.

## Introduction

Critically ill children admitted to intensive care units (ICUs) range from 75 to 166 per 100,000 children-year and respiratory disease, infectious disease, congenital disease, and perioperative care are common causes of admission [1–4]. ICU admission is costly and limited because of the high requirement for medical resources [5–7]. In developing countries, a shortage of ICU infrastructure is the main barrier to ICU admission [6, 8]. In developed countries, individual economic barriers, such as lack of health insurance, could be a problem in accessing critical care services [9, 10]. Variable types of national health insurance (NHI) systems are aiming to lower the economic barriers to healthcare access [11]. However, nationwide epidemiologic data about access to critical care services and quality of critical care under the NHI system are not well known in relation to poverty status.

Poverty could be associated with lower access to ICU admission or procedures because of its cost or geographic accessibility [12]. Decreased use of standard procedures for critically ill patients could increase mortality in impoverished patients [9]. Insufficient post-ICU care service is associated with a high readmission rate since many ICU survivors discharge with sequelae [13, 14]. Studies have shown health disparities in impoverished children, focused on a few specific critical conditions [15–18]. However, standard critical care for children aged between 1 month and 18 years is delivered in the pediatric ICUs by pediatric critical care specialists.

Therefore, comprehensive pediatric ICU data on poverty is required to make policies and to decrease health disparities. Our study aims to evaluate differences in (1) the incidence of ICU admission, (2) ICU management outcomes, and (3) readmission rates after ICU discharge between those who were in poverty and those who were not.

## Methods

### Study population and design

We conducted a retrospective cohort study using the national database from Health Insurance Review and Assessment (HIRA), a central office in the Korean Ministry of Health. Korea has a single-payer national health system. The Korean NHI covers approximately 97% of Koreans, and the remaining 3% of Koreans who cannot afford national insurance are covered by the Medical Aid Program (MAP) [19]. MAP is a public assistance program to protect socially disadvantaged or low-income family whose household income is lower than 40% of the median Korean household income (example: for 1-person family < 0.4 x $1302 = $521 per month and for 4-person family < 0.4 x $3519 = $1407 per month). In this study, we defined the poverty group as the MAP beneficiaries. The HIRA service reviews claims submitted for reimbursement to Korean NHI and MAP. Therefore, the HIRA database includes virtually all ICU admissions (including private hospitals) of all patients < 18 years of age in Korea between August 1, 2009, and September 30, 2014. We excluded patients younger than < 28 days of age because their healthcare delivery system (neonatal ICU) and insurance policies differed from those of older pediatric patients.

From this cohort, the analysis was restricted to subjects for whom information was available at 1 year before admission (wash-out period for new admission incidence) and 1 year after discharge (follow-up data for readmission). Therefore, we included pediatric admissions to an ICU between August 2010 and September 2013 (*N* = 17,927). Then we excluded patients who changed insurance status during ICU admission (*n* = 34) because their poverty exposure is ambiguous. The final sample size was 17,893 patients who were admitted between August 1, 2010, and September 30, 2013 (Additional file 1). The study was reviewed by the Institutional Review Board of Samsung Medical Center (# 2019–07-114), and informed consent was waived because we only accessed de-identified administrative data that had been previously collected. Detailed information regarding this study can be found in a previous study [1].

### Study variables

We identified ICU admissions using the claim codes that hospitals submit for cost claims for ICU management of in-hospital stays to HIRA (codes AJ100-AJ590900). We considered ICU stays during the same hospitalization as a single ICU admission. Similarly, we considered the claim codes of hospital stay separated by the same or less than 1 day as the same hospital admission. Diagnostic codes are based on the Korean Classification of Diseases 6th edition, which is the modified version of the International Classification of Diseases 10th revision adapted for use in the Korean health system [20].

Study outcome variables were in-hospital mortality, hospital readmission, readmission to the ICU, or emergency room visit within 3 months after discharge. In-hospital mortality was defined as death before discharge. Readmission was defined as an admission after hospital discharge separated by more than 1 day. To obtain readmission information, we linked study participants’ personal identification numbers to the 2010–2014 inpatient databases.

Confounding variables that could reflect the severity of disease or affect ICU admission were collected using the Korean NHI database and claim codes (Data on procedures, prescriptions, and demographic characteristics). Procedure claim codes were invasive mechanical ventilation (Korean NHI procedure codes M5857, M5858, M5860), transplantation (Q8040-Q8050, Q8140-Q8150, Q8080, Q8101-Q8103, and R3280), hemodialysis (O7020, O7062, O7051–7054), and extracorporeal membrane oxygenation (O1901-O1904). We collected vasopressor use data such as dobutamine, dopamine, epinephrine, and norepinephrine for more than 2 days, using Korean drug and anatomical therapeutic chemical codes (148201BIJ, 38900BIJ, 148701BIJ, 148702BIJ, 429500BIJ, 152601BIJ, and 203101BIJ) [21].

Hospitals were classified according to the number of hospital beds and the number of specialties as defined by the Korean Health Law [22]. A hospital is defined as a healthcare institution with more than 30 inpatient beds. A general hospital is a hospital with more than 100 beds and more than seven specialty departments. A tertiary hospital is a general hospital with more than 20 specialty departments that serves as a teaching hospital.

### Statistical analysis

We obtained population estimates for each year of age, sex, and calendar year from the NHI Service based on our definition of poverty. All analyses were conducted separately for the poverty and non-poverty groups. We calculated age-standardized ICU admission rates and mortality by the direct method [23] using the standard Korean population from Korean Statistical Information Service [24]. Mean with standard deviation or median with interquartile range were used to describe the distribution of continuous variables. We used the chi-square test and Student’s t-tests to compare categorical and continuous variables, respectively.

We used multivariable logistic regression analysis to compare the risk of in-hospital mortality between the poverty and non-poverty groups. Odds ratios with 95% confidence intervals (CI) were estimated using the model. Since patient’ demographics, diagnostic character, and treatment requirements could affect disease severity and in-hospital mortality, we adjusted for age, gender, primary diagnosis, treatment requirements (vasopressor, extracorporeal membrane oxygenation, and mechanical ventilation). Furthermore, we used hospitals as a stratification factor in the logistic regression model because patients could be clustered by the hospital. To standardize age distribution, we also performed an age and sex-weighted logistic regression for in-hospital mortality using inverse probability of treatment weighting. We estimated hazard ratios (HRs) with 95% CI for the cumulative incidence of readmission, readmission to the ICU, and emergency room (ER) visits associated with poverty after adjusting for demographic, diagnostic, and severity associated factors. We conducted subgroup analysis according to the use of mechanical ventilation to evaluate the poverty effect in subgroups with different mortality rates for possible effect modification [25]. In the subgroup analysis, the same logistic regression model was used except for adjustment of mechanical ventilation.

We considered a *p*-value < 0.05 to be significant. Statistical analyses were performed using SAS® Visual Analytics (SAS Institute Inc., USA) and STATA version 14 (StataCorp LP, College Station, TX, USA).

## Results

**Fig. 1:**
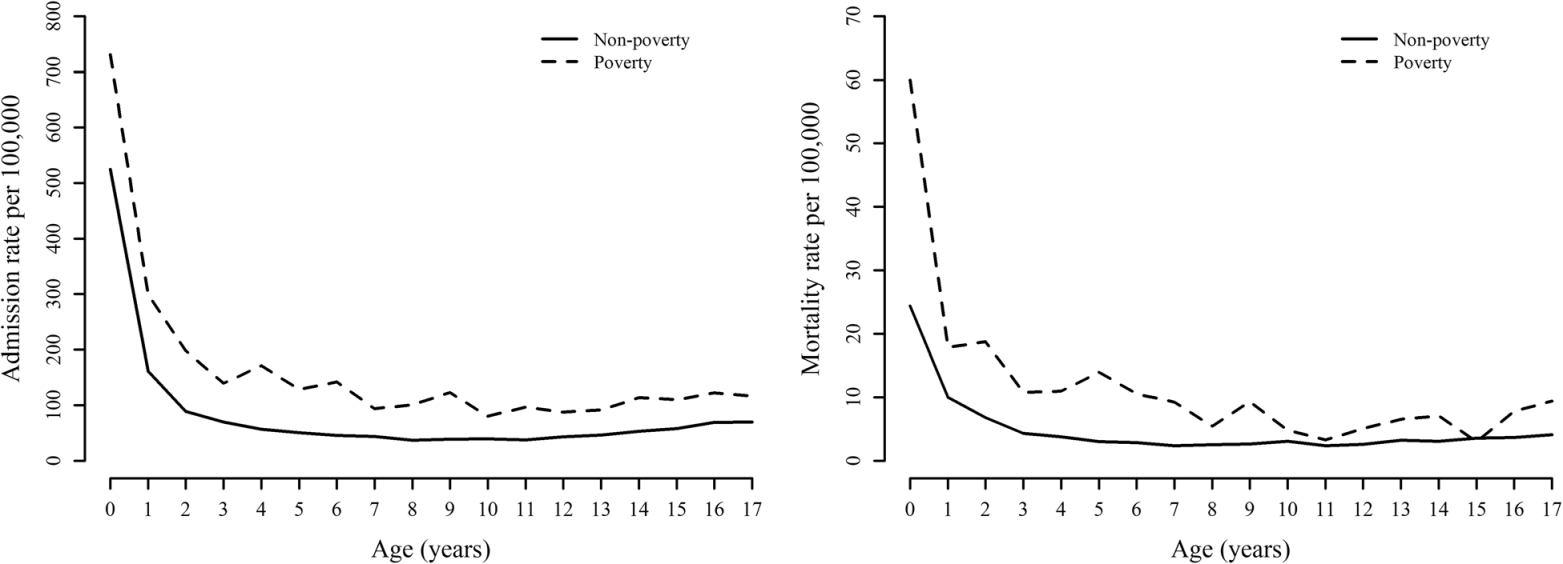
Age-standardized admission rate to intensive care units (left) and age-standardized mortality rate (right) according to poverty status by age

The mean (standard deviation) age of study patients was 7.3 (6.1) years, and 57.9% were male (*p =* 0*.145*). Among 17,893 patients, 1153 patients (6.4%) were in poverty (Table 1). From August 2010 to September 2013, the age-standardized ICU admission rate was 82.4 per 100,000 person-years (80.2 per 100,000 person-years in non-poverty patients vs. 126.9 per 100,000 person-years in poverty patients). Poverty patients had higher ICU hospitalization rates at all ages, and rates were especially high among younger patients (Fig. 1). Compared to non-poverty patients, those who were in poverty were older (7.1 vs. 10.9 years; *p* < 0.001) (Table 1). Patients in the poverty group were more likely to be admitted with injuries (22.9% vs. 17.8%), neoplasms (13.8% vs. 9.3%), neurologic diseases (10.7% vs. 9.1%), and infectious diseases (3.0% vs. 2.3%) than the non-poverty group. While 39.8 and 67.4% of ICU admissions occurred in tertiary hospitals among the poverty and non-poverty groups, respectively, and rates of mechanical ventilation and vasopressor use were higher in the non-poverty group than the poverty group (Table 1).

**Table 1:** Characteristics of pediatric patients in intensive care units according to poverty status

Variables	Overall	Non-poverty	Poverty	***p***-value
	(***N*** = 17,893)	(***n*** = 16,740)	(***n*** = 1153)	
**Age (years)**	7.3 (6.1)	7.1 (6.1)	10.9 (5.4)	< 0.001
**Age groups**				< 0.001
Infants, < 1 year	4571 (25.5)	4476 (26.7)	95 (8.2)	
Children, 1–11 years	7283 (40.7)	6894 (41.2)	389 (33.7)	
Adolescents, 12–17 years	6039 (33.8)	5370 (32.1)	669 (58.0)	
**Sex**				0.145
Male	10,357 (57.9)	9666 (57.7)	691 (59.9)	
Female	7536 (42.1)	7074 (42.3)	462 (40.1)	
**Type of hospital**				< 0.001
Tertiary hospital	11,747 (65.7)	11,288 (67.4)	459 (39.8)	
General hospital	5975 (33.4)	5300 (31.7)	675 (58.5)	
Other	171 (1.0)	152 (0.9)	19 (1.6)	
**Hospital admission type**^**a**^				0.002
Emergency department	8218 (45.9)	7638 (45.6)	580 (50.3)	
Outpatient department	9673 (54.1)	9100 (54.4)	573 (49.7)	
**Department**^**b**^				< 0.001
Medical	7481 (41.8)	6919 (41.3)	562 (48.7)	
Surgical	10,412 (58.2)	9821 (58.7)	591 (51.3)	
**Primary diagnosis**				< 0.001
Congenital anomalies	5082 (28.4)	4872 (29.1)	210 (18.2)	
Injury	3246 (18.1)	2982 (17.8)	264 (22.9)	
Respiratory disease	1945 (10.9)	1862 (11.1)	83 (7.2)	
Neoplasms	1715 (9.6)	1556 (9.3)	159 (13.8)	
Neurologic disease	1648 (9.2)	1525 (9.1)	123 (10.7)	
Circulatory disease	1531 (8.6)	1446 (8.6)	85 (7.4)	
NEC (not elsewhere classified)	622 (3.5)	556 (3.3)	66 (5.7)	
Gastrointestinal disease	464 (2.6)	439 (2.6)	25 (2.2)	
Infectious disease	426 (2.4)	391 (2.3)	35 (3.0)	
Others	1214 (6.8)	1111 (6.6)	103 (8.9)	
**Region**^**c**^				< 0.001
Seoul	8368 (46.8)	7973 (47.6)	395 (34.3)	
Metropolitan areas	3650 (20.4)	3312 (19.8)	338 (29.3)	
Rural areas	5875 (32.8)	5455 (32.6)	420 (36.4)	
**Management procedures**				
Mechanical ventilation	7624 (42.6)	7214 (43.1)	410 (35.6)	< 0.001
Vasopressors	2788 (15.6)	2646 (15.8)	142 (12.3)	0.002
CPR	835 (4.7)	770 (4.6)	65 (5.6)	0.106
Transplantation	52 (0.3)	49 (0.3)	3 (0.3)	1
Hemodialysis	456 (2.5)	423 (2.5)	33 (2.9)	0.485
ECMO	158 (0.9)	147 (0.9)	11 (1.0)	0.790

Values in the table are number (%), except for age (mean and standard deviation)

*ECMO* Extracorporeal membrane oxygenation, *CPR* Cardiopulmonary resuscitation

^a^6 (0.02%) admissions were missing in the hospital admission type

^b^Medical admissions included Pediatrics, Internal Medicine, Neurology, Neuropsychiatry, Dermatology, Rehabilitation Medicine, General, Radiology, Family Medicine, and Emergency Medicine. Surgical admissions included General Surgery, Orthopedic Surgery, Neurosurgery, Thoracic and Cardiovascular Surgery, Plastic Surgery, Ophthalmology, Otorhinolaryngology, Urology, Oral Surgery, Anesthesiology, and Obstetrics and Gynecology

^c^Regions were grouped as Seoul, metropolitan areas (Busan, Incheon, Daegu, Gwangju, Daejeon and Ulsan) and rural areas (Gyeonggi, Kangwon, Chungbuk, Chungnam, Jeonbuk, Jeonnam, Gyeongbuk, Gyeongnam, Jeju and Sejong)

The overall in-hospital mortality was 5.1% (6.0% in poverty patients and 5.1% in non-poverty patients; *p =* 0*.*223). The overall mortality of the subgroup with mechanical ventilation was 10.8%, while that of the subgroup without mechanical ventilation was 0.9%. There were 4.5 age-standardized ICU deaths per 100,000 Koreans per year (4.3 deaths per 100,000 person-years in non-poverty patients vs. 11.8 deaths per 100,000 person-years in poverty patients; Fig. 1). Multivariable analysis showed that the two groups had a similar risk of in-hospital mortality (adjusted odds ratio, 1.15; 95% CI, 0.84–1.55; *p =* 0*.*38; Table 2). In both subgroups with and without mechanical ventilation, the adjusted risk of in-hospital mortality was not statistically different between the poverty and non-poverty group. The results were similar in the inverse probability of treatment weighting analysis (odds ratio for in-hospital mortality = 1.12, 95% CI = 0.83–1.51). We mention this in the Result section as follows. Among patients who survived (*n* = 17,360), those in the poverty group were more likely to be re-admitted (adjusted HR 1.25; 95% CI 1.09–1.42) and visit the ER (adjusted HR 1.31; 95% CI 1.06–1.62) within 3 months after discharge than those in the non-poverty group were (Table 3).

**Table 2:** In-hospital mortality of pediatric patients in intensive care units according to poverty status

In-hospital mortality	No. of patients	No. of death	Unadjusted OR (95% CI)	*p*-value	Adjusted OR (95% CI)	*p*-value
Overall						
Non-poverty	16,740	850 (5.1)	*Reference*		*Reference*	
Poverty	1153	69 (6.0)	1.09 (0.85–1.42)	0.479	1.15 (0.84–1.55)	0.384
Non-mechanical ventilation						
Non-poverty	9526	88 (0.9)	*Reference*		*Reference*	
Poverty	743	9 (1.2)	1.31 (0.65–2.63)	0.446	1.71 (0.83–3.52)	0.145
Mechanical ventilation						
Non-poverty	7214	762 (10.6)	*Reference*		*Reference*	
Poverty	410	60 (14.6)	1.06 (0.78–1.44)	0.698	1.05 (0.75–1.47)	0.776

*OR* odds ratio, *CI* confidence interval

Model was adjusted for age, gender, primary diagnosis (congenital anomalies, injury, respiratory disease, neoplasms, neurologic disease, circulatory disease, NEC (not elsewhere classified), gastrointestinal disease and infectious disease), and treatment requirements (vasopressor, extracorporeal membrane oxygenation, and mechanical ventilation)

Mechanical ventilation was adjusted only in the overall analysis

*P*-for interaction was 0.588

**Table 3:** Post-ICU discharge outcomes according to poverty status (*N* = 17,360)

3 months after ICU discharge	Unadjusted HR (95% CI)	***p***-value	Adjusted HR (95% CI)	***p***-value
Overall				
Re-admission	1.12 (0.97–1.30)	0.130	**1.25 (1.09–1.42)**	**<.001**
Re-admission to the ICU	1.15 (0.88–1.51)	0.296	1.31 (0.98–1.74)	0.060
Emergency room visit	1.14 (0.90–1.43)	0.287	**1.31 (1.06–1.62)**	**0.011**
Non-mechanical ventilation				
Re-admission	0.93 (0.78–1.11)	0.411	1.13 (0.96–1.33)	0.134
Re-admission to the ICU	1.01 (0.75–1.36)	0.954	1.30 (0.94–1.80)	0.119
Emergency room visit	1.03 (0.80–1.31)	0.844	**1.26 (1.00–1.59)**	**0.048**
Mechanical ventilation				
Re-admission	1.57 (1.29–1.91)	<.001	**1.42 (1.17–1.73)**	**<.001**
Re-admission to the ICU	1.47 (0.98–2.20)	0.064	1.31 (0.86–1.98)	0.210
Emergency room visit	1.37 (1.00–1.89)	0.050	**1.35 (1.00–1.83)**	**0.049**

*ICU* intensive care unit, *HR* hazard ratio, *CI* confidence interval

Model was adjusted for age, gender, primary diagnosis (congenital anomalies, injury, respiratory disease, neoplasms, neurologic disease, circulatory disease, NEC (not elsewhere classified), gastrointestinal disease and infectious disease), and treatment requirements (vasopressor, extracorporeal membrane oxygenation, and mechanical ventilation)

Mechanical ventilation was adjusted only in the overall analysis

*P*-for interaction 0.002, 0.48, and 0.40 with re-admission, re-admission to ICU, and emergency room visit

## Discussion

In this representative population-based cohort, we showed that the age-standardized population-based ICU admission rate of children in poverty (income of their household < 0.4 x median household income of Korea) was 1.6 (126.9/80.2) times higher than that of children not in poverty. The age-standardized population-based mortality rate of children in poverty was 2.7 (11.8/4.3) times higher than that of children not in poverty. The adjusted in-hospital mortality of critically ill children admitted to the ICU was not different between the poverty and non-poverty groups.

The higher age-standardized population-based ICU admission in the poverty group was contrary to the concept that low income is associated with limited access to critical care services [9, 10]. Instead, our admission rate was similar to a study result from England and Wales (least deprived quintile of the population: 65/100,000 vs. most deprived quintile of the population: 124/100,000 per year) [3]. A high incidence of critical illness can cause a high ICU admission rate. Therefore our result might be driven by the poor pre-hospital health status of the poverty group. In previous studies, the severity of illness at ICU admission was higher in uninsured patients [9, 26], which implies worse health status at ICU admission. Poverty could affect pre-hospital health status in various ways. Mechanisms related to poor pre-hospital health status could be driven by individual characteristics (inadequate health behavior, lack of parental education, delayed presentation to primary care) or community origin (unhealthy environmental factors, low vaccination rate) [12, 27]. Difficulties in access to primary care and preventive health services may contribute to the increasing severity of illness and organ failure on hospital presentation [12]. Although the ICU admission rate of the poverty group was higher, we could not ensure that there is no disparity in ICU accessibility for a life-threatening condition. However, our study results imply that improvement of pre-hospital health status may have policy priority over lowering the barrier to ICU admission under the current NHI system.

In addition to ICU access rate, quality of ICU care represented as a mortality rate is an important health disparity issue. The high standardized mortality rate in the impoverished population highlights a critical public health problem (Fig. 1), which means a child in an impoverished family has a high risk of death. However, once children were admitted to ICU, the difference in mortality rates between the two groups was not significant when we adjusted for demographics, hospital factors, and management procedures. The result of the adjusted analysis was similar in the high mortality subgroup (mechanical ventilation) and low mortality subgroup (non-mechanical ventilation). These similar adjusted mortality rates between the two groups imply that there was no significant overall disparity in critical care quality according to poverty. Therefore, the high standardized mortality rate of the impoverished population might be driven by the high admission rate of the poverty group rather than from a high in-hospital mortality rate. However, we need to be cautious in saying there were no healthcare disparities in the ICU. The overall in-hospital mortality rate of children might be too low to identify a disparity compared to adult patients [1, 28]. Furthermore, healthcare disparities might exist in some subgroups of ICU patients.

One of the possible causes of healthcare disparity is the disparity in resource use [9]. The low use of mechanical ventilation (35.6% vs. 43.1) and vasopressor (12.3% vs. 15.8%) in the poverty group might raise suspicion of a passive attitude toward treating patients in poverty. However, the rates of mechanical ventilation and vasopressor use were different by age group in a previous study [1]. The age distribution of the poverty group was different from that of the overall population, possibly due to the different age distribution of parents under MAP from the general population. When we stratified age into 3 groups (infant, children, adolescent), there were no statistically significant differences in the incidence of mechanical ventilation and vasopressors according to poverty status (Additional file 2). In addition, the incidence of other resource-heavy procedures such as transplantation, extracorporeal membrane oxygenation, and hemodialysis was similar between the poverty and non-poverty groups in an NHI system (Table 1) (Additional file 2). These findings are contrary to the reported socioeconomic disparities in transplantation [29–31]. The difference might originate from the different health insurance systems.

Although medical resource use in ICU was not definitely different according to poverty, insufficient post-ICU care was another concern of healthcare disparity in impoverished patients. In our study, ER visits and readmission rates after ICU discharge were higher in impoverished patients. Previous studies reported that a low-socioeconomic state is associated with a high number of ER visits [32, 33]. .Low income is also associated with a high readmission rate [17, 34, 35]. It is possible that impoverished patients cannot visit outpatient clinics early phase of disease because of making money and visit the emergency room only after disease aggravation. Another possible mechanism is that frequent ER visits of the deprived population could be caused by their severe health state [36]. Although readmission rate and ER visits could be increased in severely ill patients, the poverty group of this study showed a higher risk of ER visits and readmission after severity adjustment. Therefore, we need to study the adequacy of post-ICU discharge clinics for impoverished patients.

There are some limitations to this study. First, this study was conducted in a single country where a National Healthcare Insurance system covers the impoverished with a MAP. Thus virtually no one is uninsured population. Our findings are more likely to apply to countries with a National Health Insurance system and may yield different results according to insurance status. Second, we could not use physiologic parameters or laboratory findings to evaluate the severity of illness. However, we adjusted for illness severity using the different mortality rates for the primary diagnosis, hospital factors, and treatment requirements, according to a previous study [1]. Third, we could not evaluate important demographic factors such as parent occupation and education, number of siblings, size of households as well as various barriers to ICU care. Forth, we included only new admission to ICU for comparison of mortality and readmission risk. Therefore our admission rates did not reflect the burden of multiple admissions (28.5% admissions in the poverty group vs. 22.5% admissions in the non-poverty group). However, the finding that the standardized ICU admission rate in the poverty group is higher than that of the non-poverty group did not change with the inclusion of readmissions.

Despite these limitations, we included all hospitals where pediatric patients are admitted to the ICU, except for rare possible administrative losses of insurance claims, which allowed us to calculate the population-based admission rate and mortality rate. Previous studies conducted at select hospitals were not free from selection bias of hospitals [16, 26, 37]. Utilization of high-volume or top-ranked (low mortality) hospitals differed according to poverty status [38, 39], and data from these renowned hospitals could bias admission rates of low-income patients.

Our findings also have implications for reducing disparities in mortality in patients living in poverty. The NHI system with a single-payer might work well to provide healthcare in the ICU to impoverished patients without disparity, but lowering economic barriers to hospital healthcare by the NHI might not be sufficient to reduce mortality of impoverished patients. Policies to improve the pre-hospital health status of patients in poverty are required to decrease ICU admission and population-based mortality.

## Conclusion

Under the NHI system, the disparity in pediatric critical care outcomes according to poverty is not definite, but the healthcare disparity in pre- and post-hospital care is a concern. Further studies are required to improve pre- and post-hospital healthcare quality of impoverished children.

## Supplementary Information

**Additional file 1: Figure S1.** Flow chart of patient selection with inclusion and exclusion

**Additional file 2: Table S1.** Characteristics of pediatric patients in intensive care units according to poverty status by age groups.

## Data Availability

The researchers can access on the intranet of Korean Health Insurance Review & Assessment Service through the URL http://opendata.hira.or.kr/home.do after approval of the request. The researchers can request the same periods, terms and items (claim codes) as done in this study. The authors did not have any special access privileges that others would not have.

## Abbreviations

ICU: Intensive care unit
NHI: National Health Insurance
MAP: Medical Aid Program
HIRA: Health Insurance Review and Assessment
CI: Confidence interval
HR: Hazard ratio
ER: Emergency room
SES: Socioeconomic status
