# Supplementary figures and images for: Health disparities of critically ill children according to poverty: the Korean population-based retrospective cohort study
Source: BMC Public Health. 2021 Jun 30;21:1274. doi: 10.1186/s12889-021-11324-4 (PMC8243750; doi:10.1186/s12889-021-11324-4)

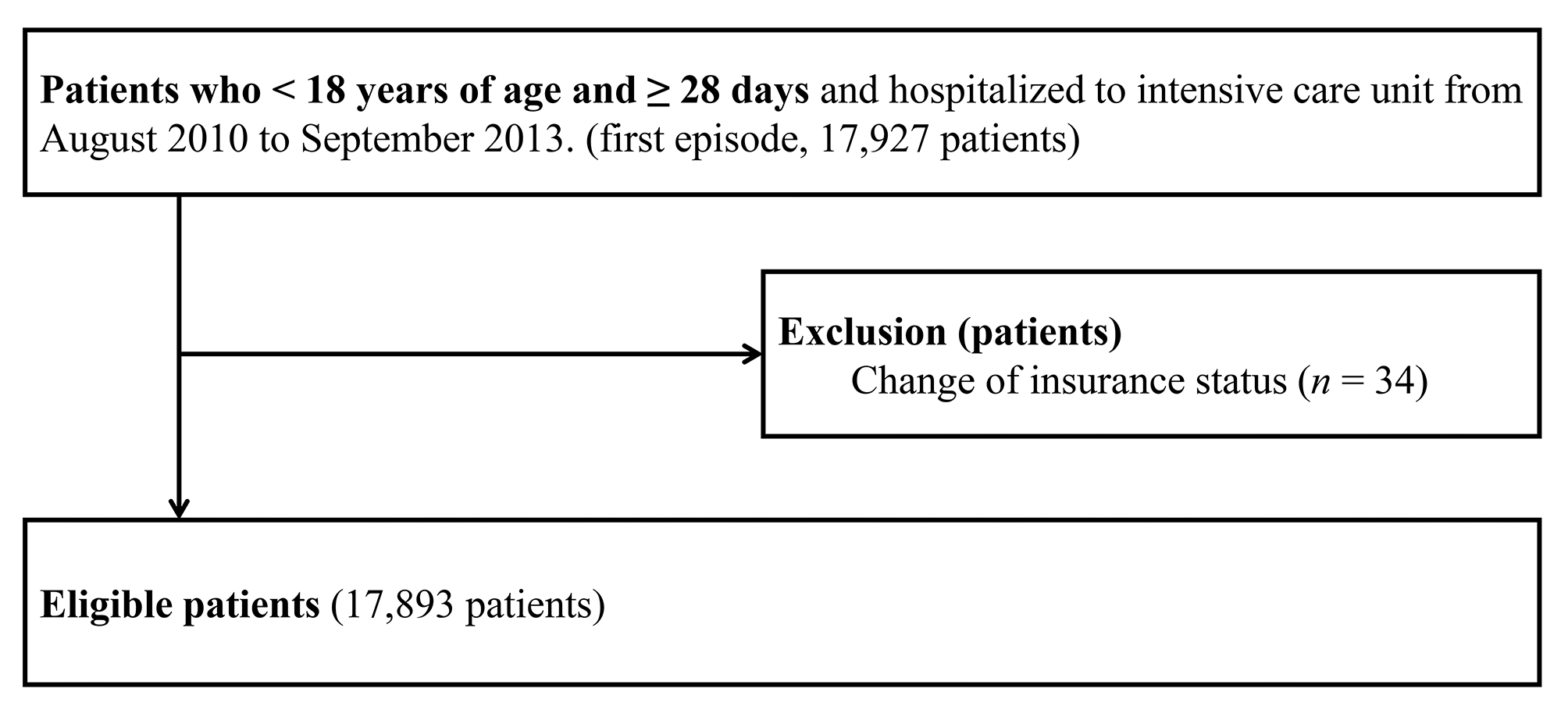

Supplement: Supplementary file 1 — Additional file 1: Figure S1. Flow chart of patient selection with inclusion and exclusion [file 12889_2021_11324_MOESM1_ESM.tif]
