# Supplementary material for: Health disparities of critically ill children according to poverty: the Korean population-based retrospective cohort study
Source: BMC Public Health. 2021 Jun 30;21:1274. doi: 10.1186/s12889-021-11324-4 (PMC8243750; doi:10.1186/s12889-021-11324-4)
Supplement: Supplementary file 2 — Additional file 2: Table S1. Characteristics of pediatric patients in intensive care units according to poverty status by age groups. [file 12889_2021_11324_MOESM2_ESM.docx]

| **Supplemental Table 1**. Characteristics of pediatric patients in intensive care units according to poverty status by age groups. | | | | | | | | | |
| --- | --- | --- | --- | --- | --- | --- | --- | --- | --- |
| **Variables** | **Infants, <1 year** | | | **Children, 1–11 years** | | | **Adolescents, 12–17 years** | | |
|  | **Poverty status** | | | **Poverty status** | | | **Poverty status** | | |
|  | **No** | **Yes** |  | **No** | **Yes** |  | **No** | **Yes** |  |
|  | **(*n* = 4476)** | **(*n* = 95)** | ***p*-value** | **(*n* = 6894)** | **(*n* = 389)** | ***p*-value** | **(*n* = 5370)** | **(*n* = 669)** | ***p*-value** |
| **Sex** |  |  | 0.801 |  |  | 0.35 |  |  | 0.83 |
| Male | 2486 (55.5) | 54 (56.8) |  | 3874 (56.2) | 228 (58.6) |  | 3306 (61.6) | 409 (61.1) |  |
| Female | 1990 (44.5) | 41 (43.2) |  | 3020 (43.8) | 161 (41.4) |  | 2064 (38.4) | 260 (38.9) |  |
| **Type of hospital** |  |  | 0.004 |  |  | <0.001 |  |  | <0.001 |
| Tertiary hospital | 3283 (73.3) | 61 (64.2) |  | 4894 (71.0) | 181 (46.5) |  | 3111 (57.9) | 217 (32.4) |  |
| General hospital | 1183 (26.4) | 32 (33.7) |  | 1945 (28.2) | 203 (52.2) |  | 2172 (40.4) | 440 (65.8) |  |
| Other | 10 (0.2) | 2 (2.1) |  | 55 (0.8) | 5 (1.3) |  | 87 (1.6) | 12 (1.8) |  |
| **Hospital admission type^c^** |  |  | 0.436 |  |  | 0.9 |  |  | 0.814 |
| ER | 1433 (32.0) | 34 (35.8) |  | 3266 (47.4) | 186 (47.8) |  | 2939 (54.7) | 363 (54.3) |  |
| OPD | 3043 (68.0) | 61 (64.2) |  | 3627 (52.6) | 206 (53) |  | 2430 (45.3) | 306 (45.7) |  |
| **Department*** |  |  | 0.007 |  |  | 0.045 |  |  | <0.001 |
| Medical | 1924 (43.0) | 54 (56.8) |  | 2940 (42.6) | 186 (47.8) |  | 2055 (38.3) | 322 (48.1) |  |
| Surgical | 2552 (57.0) | 41 (43.2) |  | 3954 (57.4) | 203 (52.2) |  | 3315 (61.7) | 347 (51.9) |  |
| **Primary diagnosis** |  |  | 0.077 |  |  | <0.001 |  |  | <0.001 |
| Congenital anomalies | 2616 (58.4) | 47 (49.5) |  | 1714 (24.9) | 105 (27) |  | 542 (10.1) | 58 (8.7) |  |
| Injury | 181 (4.0) | 6 (6.3) |  | 1150 (16.7) | 49 (12.6) |  | 1651 (30.7) | 209 (31.2) |  |
| Respiratory disease | 175 (3.9) | 2 (2.1) |  | 913 (13.2) | 22 (5.7) |  | 774 (14.4) | 59 (8.8) |  |
| Neoplasms | 260 (5.8) | 5 (5.3) |  | 872 (12.6) | 72 (18.5) |  | 424 (7.9) | 82 (12.3) |  |
| Neurologic disease | 584 (13.0) | 12 (12.6) |  | 675 (9.8) | 50 (12.9) |  | 266 (5.0) | 61 (9.1) |  |
| Circulatory disease | 174 (3.9) | 5 (5.3) |  | 597 (8.7) | 21 (5.4) |  | 675 (12.6) | 59 (8.8) |  |
| NEC (not elsewhere classified) | 96 (2.1) | 1 (1.1) |  | 210 (3) | 22 (5.7) |  | 250 (4.7) | 43 (6.4) |  |
| Gastrointestinal disease | 111 (2.5) | 6 (6.3) |  | 243 (3.5) | 11 (2.8) |  | 85 (1.6) | 8 (1.2) |  |
| Infectious disease | 111 (2.5) | 6 (6.3) |  | 168 (2.4) | 13 (3.3) |  | 112 (2.1) | 16 (2.4) |  |
| Others | 168 (3.8) | 5 (5.3) |  | 352 (5.1) | 24 (6.2) |  | 591 (11) | 74 (11.1) |  |
| **Region^b^** |  |  | <0.001 |  |  | <0.001 |  |  | <0.001 |
| Seoul | 2318 (51.8) | 40 (42.1) |  | 3526 (51.1) | 158 (40.6) |  | 2129 (39.6) | 197 (29.4) |  |
| Metropolitan areas | 820 (18.3) | 32 (33.7) |  | 1333 (19.3) | 121 (31.1) |  | 1159 (21.6) | 185 (27.7) |  |
| Rural areas | 1338 (29.9) | 23 (24.2) |  | 2035 (29.5) | 110 (28.3) |  | 2082 (38.8) | 287 (42.9) |  |
| **Management procedures** |  |  |  |  |  |  |  |  |  |
| Mechanical ventilation^a^ | 3049 (68.1) | 63 (66.3) | 0.709 | 2734 (39.7) | 164 (42.2) | 0.327 | 1431 (26.6) | 183 (27.4) | 0.697 |
| Vasopressors | 1543 (34.5) | 29 (30.5) | 0.423 | 1057 (15.3) | 61 (15.7) | 0.853 | 669 (12.5) | 77 (11.5) | 0.482 |
| CPR | 228 (5.1) | 14 (14.7) | <0.001 | 321 (4.7) | 14 (3.6) | 0.333 | 221 (4.1) | 37 (5.5) | 0.088 |
| Transplantation | 5 (0.1) | 0 (0) | 1 | 22 (0.3) | 1 (0.3) | 1 | 22 (0.4) | 2 (0.3) | 1 |
| Hemodialysis | 84 (1.9) | 1 (1.1) | 1 | 162 (2.3) | 8 (2.1) | 0.709 | 177 (3.3) | 24 (3.6) | 0.692 |
| ECMO | 37 (0.8) | 2 (2.1) | 0.194 | 60 (0.9) | 3 (0.8) | 1 | 50 (0.9) | 6 (0.9) | 0.931 |

Values in the table are number (%), except for age (mean and standard deviation).

ER: Emergency room; OPD: Outpatient department; CPR: Cardiopulmonary resuscitation; ECMO: Extracorporeal membrane oxygenation.

^a^Medical admissions included Pediatrics, Internal Medicine, Neurology, Neuropsychiatry, Dermatology, Rehabilitation Medicine, General, Radiology, Family Medicine, and Emergency Medicine. Surgical admissions included General Surgery, Orthopedic Surgery, Neurosurgery, Thoracic and Cardiovascular Surgery, Plastic Surgery, Ophthalmology, Otorhinolaryngology, Urology, Oral Surgery, Anesthesiology, and Obstetrics and Gynecology

^b^Regions were grouped as Seoul, metropolitan areas (Busan, Incheon, Daegu, Gwangju, Daejeon and Ulsan) and rural areas (Gyeonggi, Kangwon, Chungbuk, Chungnam, Jeonbuk, Jeonnam, Gyeongbuk, Gyeongnam, Jeju and Sejong).

^c^6 (0.02%) admissions were missing in the hospital admission type.
